# The different effect of adverse childhood experiences on Theory of Mind brain networks in schizophrenia and healthy controls

**DOI:** 10.1192/j.eurpsy.2023.810

**Published:** 2023-07-19

**Authors:** A. Pelucchi, F. Calesella, M. Bechi, R. Cavallaro, S. Poletti, B. Vai, F. Benedetti

**Affiliations:** 1 Psychiatry and Clinical Psychobiology, Division of Neuroscience, IRCCS Ospedale San Raffaele; 2 University Vita-Salute San Raffaele; 3 Department of Clinical Neurosciences, IRCCS San Raffaele Scientific Institute Hospital, Milano, Italy

## Abstract

**Introduction:**

Deficit in Theory of Mind (ToM) is a core feature of schizophrenia (SZ), while adverse childhood experiences (ACEs) can contribute to worsen ToM abilities through their effect on brain functioning, structure and connectivity.

**Objectives:**

Here, we investigated the effects of ACEs on brain functional connectivity (FC) during an affective and cognitive ToM task (AToM, CToM) in healthy control (HC) and SZ, and whether FC can predict the performance at the ToM task and patients’ symptoms severity.

**Methods:**

The sample included 26 HC and 33 SZ. In an fMRI session, participants performed a ToM task targeting affective and cognitive domains. Whole-brain FC patterns of local correlation (LC) and multivariate pattern analysis (MVPA) were extracted. The significant MVPA clusters were used as seeds in further seed-based connectivity analyses. Second-level analyses were modelled to investigate the interaction between ACEs, the diagnosis, and the task, corrected for age, sex, and equivalent doses of chlorpromazine (p<0.05 FWE). FC values significantly affected by ACEs (Risky Family Questionnaire) were entered in a cross-validated LASSO regression predicting symptoms severity (Positive and Negative Syndrome Scale, PANSS) and task performance measures (accuracy and response time).

**Results:**

In AToM, LC showed significant different effects of ACE between HC and SZ in frontal pole, caudate and cerebellum. MVPA showed significant widespread interaction in cortico-limbic regions, including prefrontal cortex, precuneus, insula, parahippocampus, cingulate cortex, temporal pole, thalamus, and cerebellum in AToM and CToM. SBC analyses found significant target regions in the frontal pole, cerebellum, pre and postcentral gyrus, precuneus, lateral occipital cortex, angular gyrus, and paracingulate gyrus. LASSO regression predicted PANSS score (R^2^=0.49) and AToM response latency time (R^2^=0.37).

**Conclusions:**

Our findings highlighted a widespread different effect of ACEs on brain FC in ToM networks in HC and SZ. Notably, the FC in these regions is predictive of behavioral ToM performance and clinical outcomes.

**Disclosure of Interest:**

None Declared

